# Diagnostic utility of whole-body computed tomography/pan-scan in trauma: a systematic review and meta-analysis study

**DOI:** 10.1007/s10140-024-02213-5

**Published:** 2024-02-23

**Authors:** Mobina Fathi, Arshia Mirjafari, Shirin Yaghoobpoor, Milad Ghanikolahloo, Zohre Sadeghi, Ashkan Bahrami, Lee Myers, Ali Gholamrezanezhad

**Affiliations:** 1 Student Research Committee, School of Medicine, Shahid Beheshti University of Medical Sciences, Tehran, Iran; 2 Center for Minimally Invasive Therapeutics (C-MIT), University of California, Los Angeles, CA USA; 3 Department of Bioengineering, University of California, Los Angeles, CA USA; 4 Department of Radiological Sciences, David Geffen School of Medicine, University of California, Los Angeles, CA USA; 5 Terasaki Institute for Biomedical Innovation, Los Angeles, CA USA; 6 King’s College Hospital NHS Foundation Trust, London, UK; 7 Department of Radiology, Imam Khomeini Hospital Complex, Tehran, Iran; 8 Faculty of Medicine, Kashan University of Medical Science, Kashan, Iran; 9 Department of Radiology, Keck School of Medicine, University of Southern California, Los Angeles, CA USA; 10 Department of Radiology, Keck School of Medicine, University of Southern California (USC), Los Angeles, USA

**Keywords:** Whole-body CT scan, Pan-scan, Head and neck injury, Mortality rate

## Abstract

Trauma is a significant cause of mortality and morbidity. It is crucial to diagnose trauma patients quickly to provide effective treatment interventions in such conditions. Whole-body computed tomography (WBCT)/pan-scan is an imaging technique that enables a faster and more efficient diagnosis for polytrauma patients. The purpose of this systematic review and meta-analysis is to evaluate the efficacy of WBCT in diagnosing injuries in polytrauma patients. We will also assess its impact on the mortality rate and length of hospital stay among trauma centers between patients who underwent WBCT and those who did not (non-WBCT). Twenty-seven studies meeting our inclusion criteria were selected among PubMed, Scopus, Web of Science, and Google Scholar. The criteria were centered on the significance of WBCT/pan-scan application in trauma patients. Stata version 15 was used to perform statistical analysis on the data. The authors have also used *I*^2^ statistics to evaluate heterogeneity. Egger and Begg’s tests were performed to rule out any publication bias. Total of twenty-seven studies including 68,838 trauma patients with a mean age of 45.0 ± 24.7 years were selected. Motor vehicle collisions were the most common cause of blunt injuries (80.0%). Head, neck, and face injuries were diagnosed in 44% (95% CI, 0.28–0.60; *I*^2^ = 99.8%), 6% (95% CI, 0.02–0.09; *I*^2^ = 97.2%), and 9% (95% CI, 0.05–0.13; *I*^2^ = 97.1%), respectively. Chest injuries were diagnosed by WBCT in 39% (95% CI, 0.28–0.51; *I*^2^ = 99.8%), abdominal injuries in 23% (95% CI, 0.03–0.43; *I*^2^ = 99.9%) of cases, spinal injuries 19% (95% CI, 0.11–0.27; *I*^2^ = 99.4%), extremity injuries 33% (95% CI, 0.23–0.43; *I*^2^ = 99.2%), and pelvic injuries 11% (95% CI, 0.04–0.18; *I*^2^ = 97.4%). A mortality odd ratio of 0.94 (95% CI, 0.83–1.06; *I*^2^ = 40.1%) was calculated while comparing WBCT and non-WBCT groups. This systematic review and meta-analysis provide insight into the possible safety, efficacy, and efficiency of WBCT/pan-scan as a diagnostic tool for trauma patients with serious injuries, regardless of their hemodynamic status. In patients with serious injuries from trauma, whether or not there are indicators of hemodynamic instability, our recommended approach is to, wherever possible, perform a WBCT without stopping the hemostatic resuscitation. By using this technology, the optimal surgical strategy for these patients can be decided upon without causing any delays in their final care or greatly raising their radiation dose.

## Introduction

The World Health Organization database report in 2021 demonstrates that traumatic injuries are the cause for mortality in 4.4 million people worldwide each year, accounting for nearly 8% of all deaths, including road traffic crashes, suicide, homicide, war and conflict, drowning, and falls [1]. Protocols of trauma alert, which is triage-based, early evaluation of severely injured patients benefit significantly from trauma team treatment. Serious injury is defined as the Injury Severity Score (ISS) > 15; however, this score is only possible once the entire extent of injury has been determined [2, 3]. Imaging techniques that quickly assess the full extent of traumatic injuries can help direct clinical decision-making. [4].

Whole-body computed tomography (WBCT) or pan-scan is routinely used in trauma centers for detecting injuries; this tool is recognized as a comprehensive tool to rapidly access injuries in trauma patients to direct management of life-saving procedures [3, 5], evaluations of injuries not identified on the primary and secondary surveys as well as detailed evaluation of suspected injuries [6, 7]. It is important to have a complete overview of all traumatic injuries to coordinate a comprehensive treatment plan [8, 9]. WBCT can reveal occult injuries not suspected on the primary and secondary surveys, which can be of critical clinical importance [10–12]. Unlike other imaging methods, WBCT allows for an evaluation by capturing injuries from head to toe in a single evaluation [5, 13, 14]. Given the variety of trauma causes, such as motor vehicle collisions, falls, and assaults, WBCT evaluates a wide variety of injuries regardless of underlying mechanism [14, 15]. In severe blunt trauma patients who received WBCT, 30-day mortality decreased significantly, and lower 24-h mortality rates were obtained [16, 17]. WBCT can also play a vital role as an essential diagnostic method in detecting life-threatening injuries during the early resuscitation phase for patients with polytrauma [18]. Furthermore, while WBCT reduces emergency department (ED) time, its effect on hospital stay is unclear [19, 20]. Some authors found no difference in hospital length of stay (LOS) between WBCT and selective CT imaging, while others found a longer length of stay (LOS) in trauma patients with incidental CT findings [21, 22].

Whole-body CT has many benefits including imaging evaluation of the entire body and decreased acquisition time compared with selective imaging, which may lead to decreased mortality rates and length of hospital stay. This meta-analysis aims to examine the mortality rates and length of hospital stay for trauma patients who underwent WBCT. Thorough investigation of injuries to the head, neck, face, chest, abdomen, brain, spinal region, musculoskeletal system, and pelvis may also give insights into injuries that were not expected after the primary and secondary surveys. In summary, our study intends to give information about the severity of injuries, the mortality rate, the length of hospital stays, and region-specific injuries in trauma patients who underwent WBCT and shed light on the importance of employing WBCT/pan-scan to better diagnose traumatic injuries.

## Methods

### Study selection

A total of 3612 articles published before December 2023 were queried and retrieved from PubMed (1664), Scopus (1403), and Web of Science (545) databases while adhering to the Preferred Reporting Items for Systematic Review and Meta-Analysis (PRISMA) guidelines [23]. The keywords of our search query included “whole body” OR “Pan scan” OR “total body scan” AND “CT scan” AND “Trauma OR wound OR injury”. References of each article were individually inspected to ensure no overlaps/duplicate data.

### Inclusion and exclusion criteria

All studies were reviewed and filtered based on their scope, and 27 were included in the present meta-analysis. Articles discussing the clinical significance and effectiveness of human WBCT/pan-scan application for diagnosing trauma patients have been chosen in the present study. In the literature review, we found a large amount of inconsistency between papers specifying the protocols used when describing WBCT, total body CT (TBCT), or pan-scan. Pan-scan was the imaging of the head, chest, cervical spine, abdomen, and pelvis [24]. WBCT was mainly defined as the unenhanced head CT followed by contrast-enhanced CT of the chest, abdomen, pelvis, and the complete spine [25]. This was while the definition of TBCT stood like pan-scan as a non-enhanced CT scan of the head and neck, with arms alongside the trunk, followed by a contrast-enhanced CT scan of the chest, abdomen, and pelvis. [26–31]. Therefore, to cover all papers with the eligible criteria, we added all of them to our analysis. These articles were thoroughly reviewed and inspected for their setting, design, scope, and data. Studies focused on radiographic modalities differing from CT, non-immediate WBCT/pan-scan, non-emergency, and non-trauma cases were filtered out. Papers with inadequate data for the scope of this study, non-English, case reports, and review articles were excluded.

### Data extraction

The final selection of papers was screened and inspected by two authors (AM and MF), and data on the following parameters within those articles were collected for final analysis: number of patients, sex, age, ISS, mechanism of injury, mortality rate, length of hospital stay, diagnosed injury.

### Statistical analysis

**Fig. 1:**
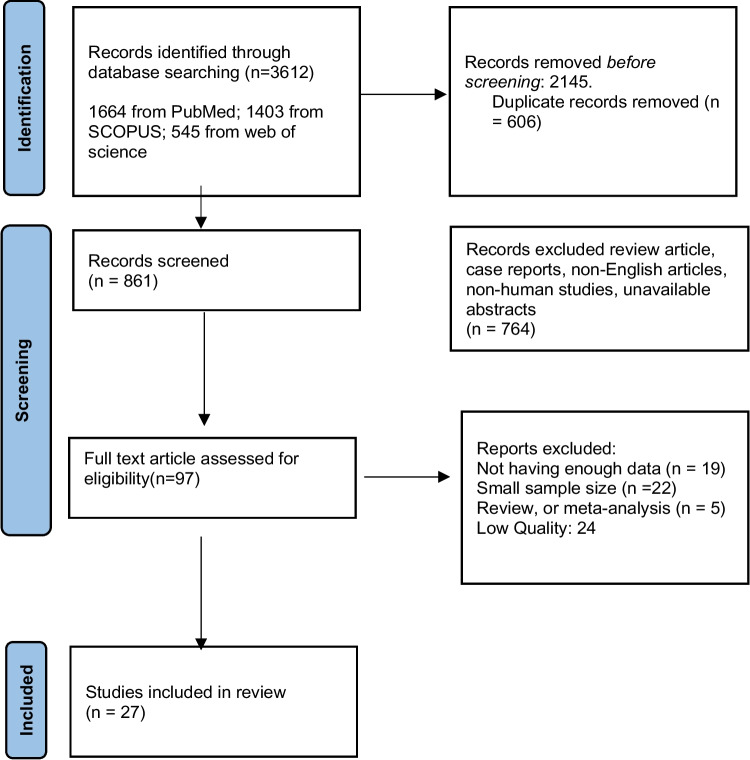
Study flow diagram

**Fig. 2:**
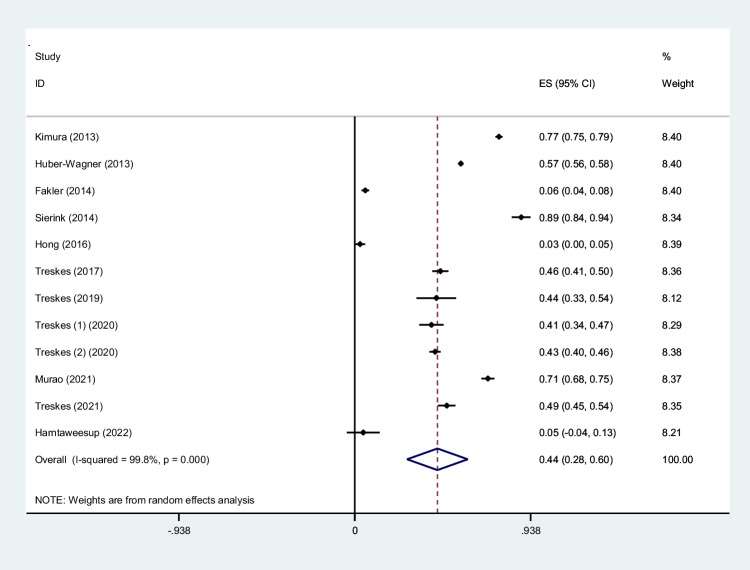
Forest plot of the prevalence of head injuries using WBCT/pan-scan among trauma patients. The weight of each paper on the meta-analysis is indicated by each parallelogram; the 95% CI is visualized by the interval within the boundaries. Literature is presented based on random effect model

**Fig. 3:**
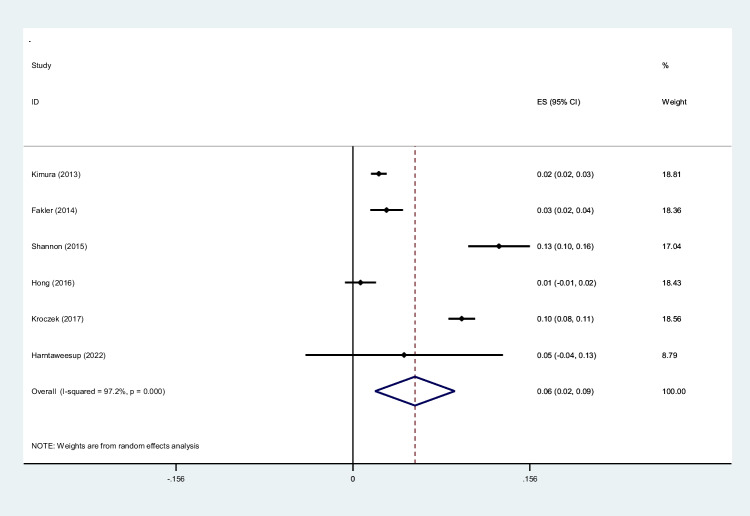
Forest plot of the prevalence of neck injuries using WBCT/pan-scan among trauma patients. The weight of each paper on the meta-analysis is indicated by each parallelogram; the 95% CI is visualized by the interval within the boundaries. Literature is presented based on random effect model

**Fig. 4:**
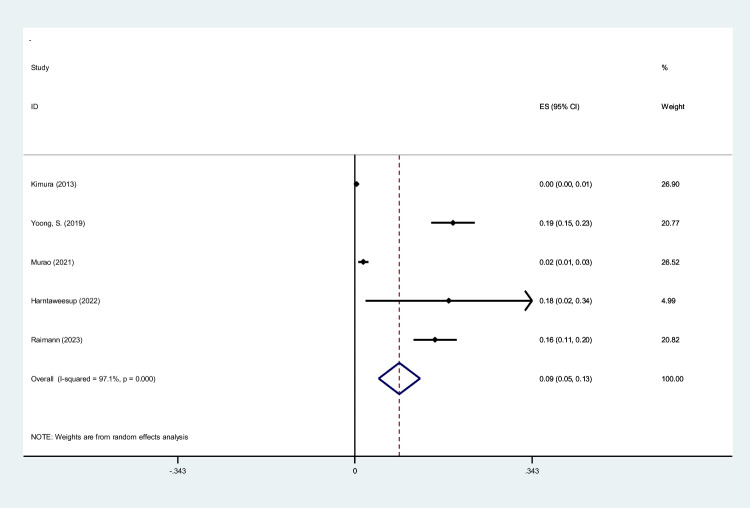
Forest plot of the prevalence of face injuries using WBCT/pan-scan among trauma patients. The weight of each paper on the meta-analysis is indicated by each parallelogram; the 95% CI is visualized by the interval within the boundaries. Literature is presented based on random effect model

**Fig. 5:**
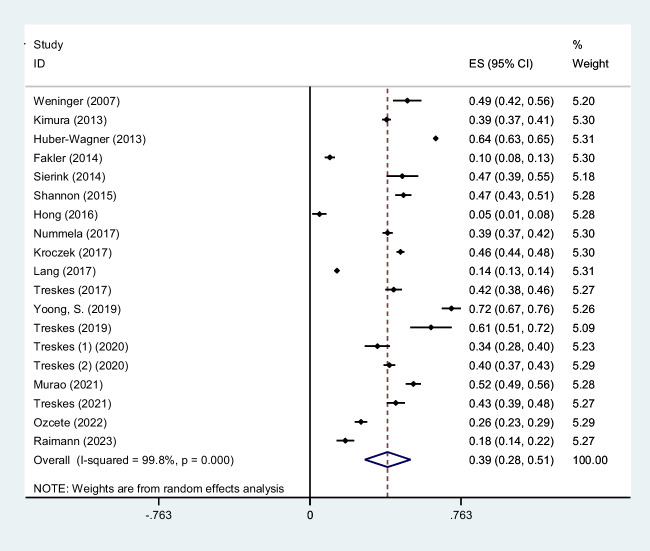
Forest plot of the prevalence of chest injuries using WBCT/pan-scan among trauma patients. The weight of each paper on the meta-analysis is indicated by each parallelogram; the 95% CI is visualized by the interval within the boundaries. Literature is presented based on random effect model

**Fig. 6:**
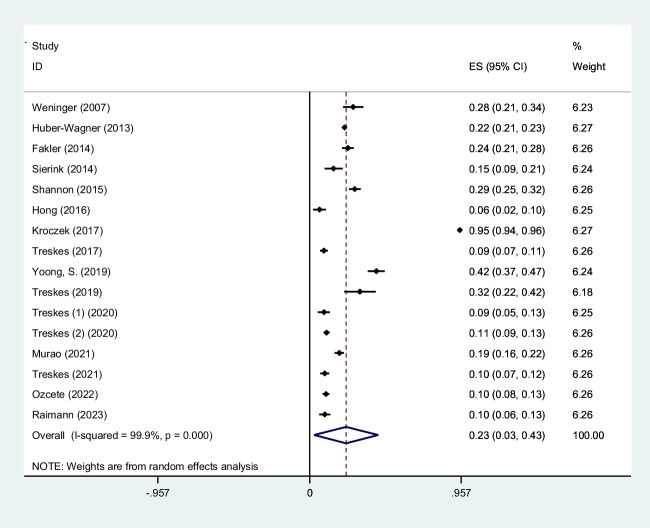
Forest plot of the prevalence of abdomen injuries using WBCT/pan-scan among trauma patients. The weight of each paper on the meta-analysis is indicated by each parallelogram; the 95% CI is visualized by the interval within the boundaries. Literature is presented based on random effect model

**Fig. 7:**
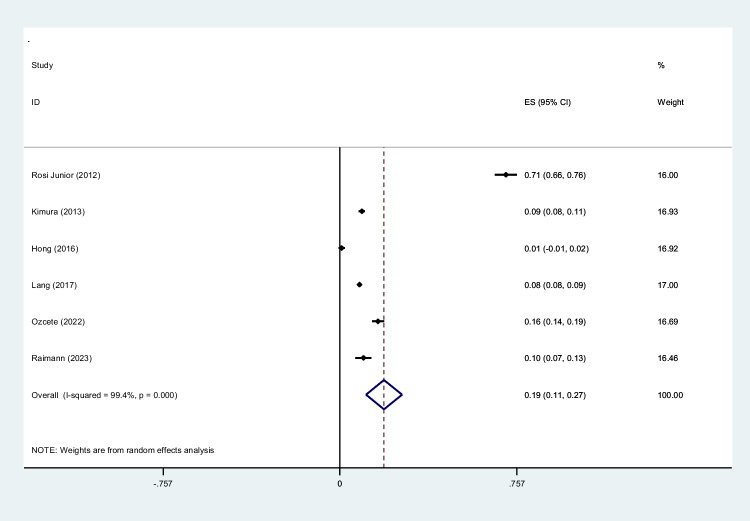
Forest plot of the prevalence of spine injuries using WBCT/pan-scan among trauma patients. The weight of each paper on the meta-analysis is indicated by each parallelogram; the 95% CI is visualized by the interval within the boundaries. Literature is presented based on random effect model

**Fig. 8:**
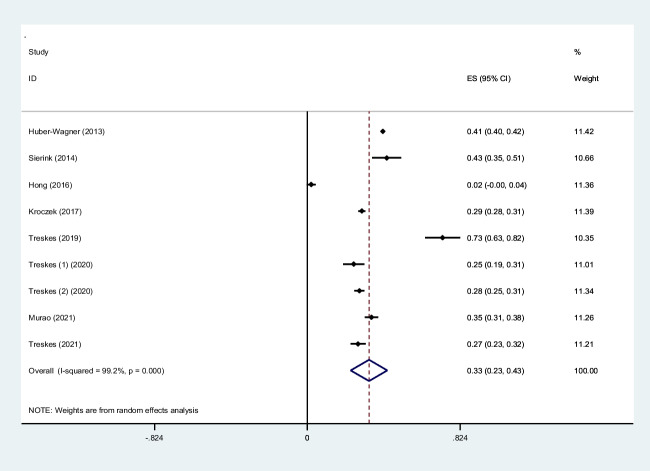
Forest plot of the prevalence of extremity injuries using WBCT/pan-scan among trauma patients. The weight of each paper on the meta-analysis is indicated by each parallelogram; the 95% CI is visualized by the interval within the boundaries. Literature is presented based on random effect model

**Fig. 9:**
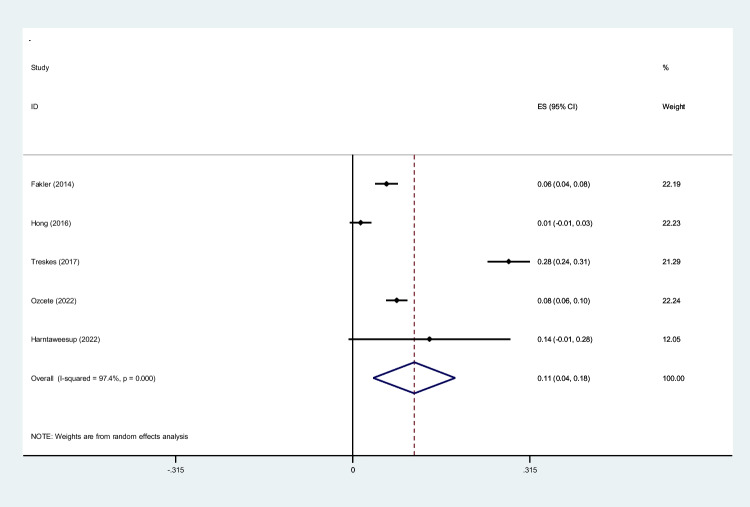
Forest plot of the prevalence of pelvic injuries using WBCT/pan-scan among trauma patients. The weight of each paper on the meta-analysis is indicated by each parallelogram; the 95% CI is visualized by the interval within the boundaries. Literature is presented based on random effect model

**Fig. 10:**
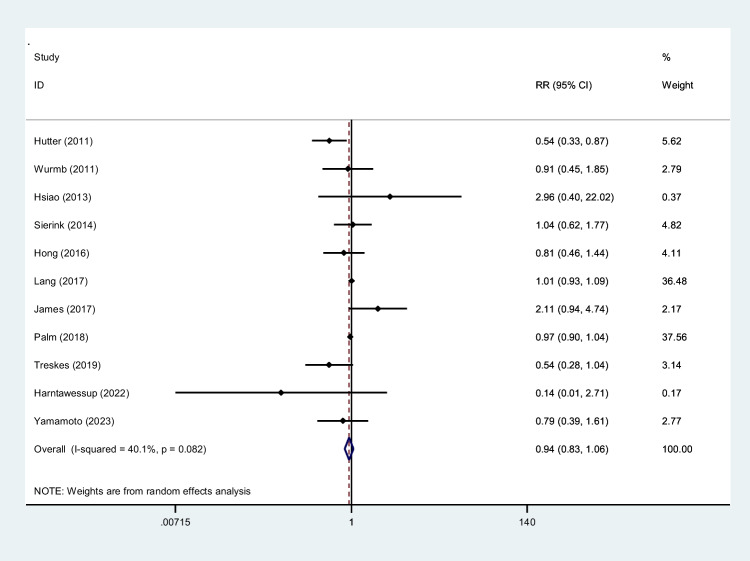
Forest plot of the odds ratio of mortality using WBCT/pan-scan among trauma patients. The weight of each paper on the meta-analysis is indicated by each parallelogram; the 95% CI is visualized by the interval within the boundaries. Literature is presented based on random effect model

To screen the prevalence of region-specific injuries in trauma cases, a meta-analysis was performed using Stata version 15 USA [32]. Subject to adequate data per region, data extraction and meta-analysis were performed. The prevalence rate was selected as the unit for the effect size in this study. The odd ratio was chosen as the effect size for mortality analysis. Data was analyzed while adhering to the random effects model. *I*^2^ statistics were used to assess heterogeneity, and values greater than 50% were flagged for high heterogeneity. Publication bias was performed quantitatively using Begg and Egger’s regression test and visualized with funnel plots (Figs. 1, 2, 3, 4, 5, 6, 7, 8, 9, and 10).

### Publication bias

**Fig. 11:**
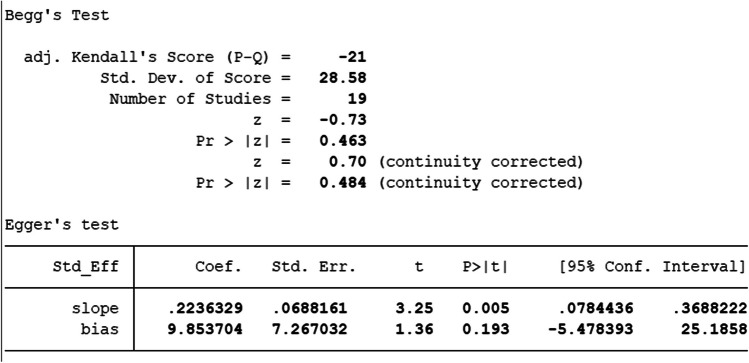
Begg and Egger’s publication bias

**Fig. 12:**
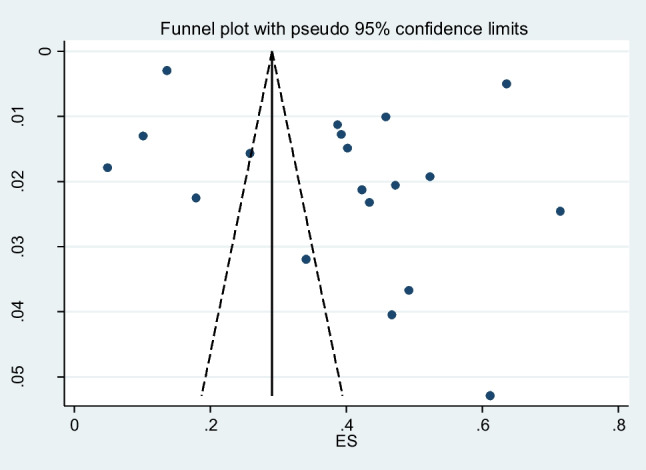
Funnel plot of the publication bias

Egger and Begg’s test [33] was performed to assess publication bias within the chosen literature; adhering to conventions, *P* < 0.05 constitutes a significant publication bias (Figs. 11 and 12). Further, a linear regression analysis containing intercept and slope parameters was performed. The formula *yi* ¼ *a* + *bxi* + *ϵi* [*i* = 1… *r* (*r* = the number of studies), *yi* = standardized estimate, *xi* = precision of studies, *ϵi* = error terms] was used to calculate the named parameters.

### Quality assessment

**Fig. 13:**
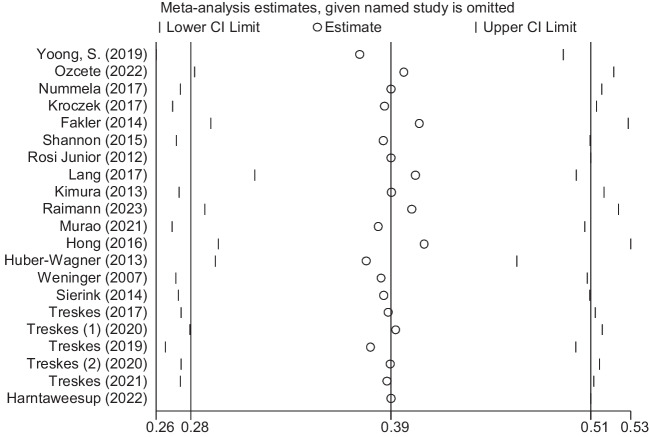
Sensitivity analysis of the included studies in the prevalence analysis

Using the Newcastle–Ottawa scale (NOS) [34], the quality of each included paper was closely examined and recorded. Employing eight separate evaluations, including “selection,” “comparability,” and “outcome,” the Ottawa checklist for the cross-sectional studies yields a correlational score to each study’s statistical power. The NOS scoring system for cross-sectional studies is as follows: Very Good Studies (9–10 points), Good Studies (7–8 points), Satisfactory Studies (5–6 points), and Unsatisfactory Studies (0 to 4 points) (Fig. 13).

## Results

### Study selection and characteristics

Initially, 3412 articles were obtained from searching databases including PubMed, Scopus, and Web of Science. 2751 records were excluded based on the title and abstract screening or being duplicates before assessing the full text. 764 out of 861 studies were filtered after elimination of non-English articles, reviews, non-available abstracts, and irrelevant to the main subject. The full texts of the 97 remaining articles were fully assessed, and 70 more studies were excluded due to unclear or insufficient data (*n* = 46) and low quality (*n* = 24). After full-text screening of the remaining articles, 27 articles were eligible and included in the meta-analysis (Fig. 1).

Adhering to PRISMA standards and conventions of meta-analysis [23], 68,838 patients were studied among the selected studies. Overall, 67.5% of the presented trauma patients studied were male. The mean age of presentation was 45.0 ± 24.7 years. Twenty studies introduced and monitored Injury Severity Score among their patients/databases as a correlational measure of positive prediction value; within these studies, the mean ISS was 22.49 ± 11.53.

### Mechanisms of injury (MOI)

Detailed categorization of trauma cases from 13 studies revealed the frequency of the mechanisms of injury among cases in the emergency room [5, 14, 28, 30, 31, 35–42]. Motor vehicle accidents (car, bicycle, scooter, pedestrian, etc.) accounted for 6167 injuries (80.0%), thereby being the leading cause of trauma among patients, followed by falls (4027, 20.0%). Penetrating and assault-related injuries were less common and counted for other mechanisms of injury 1518 (5.0%) (Table 1 and 2).

**Table 1:** Baseline characteristics in studies included in this meta-analysis

First author/date	Technique	Number of patients	IWBCT population	Control population	Male	Age (mean/SD)	ISS (mean/median)	Injury type	Frequency of injuries	MOI	MOI frequency	LOS IWBCT (days)	LOS control (days)	MR IWBCT	MR control	NOS assessment score
Raimann, 2023 [40]	WBCT	290	156	134	187	11.32	10.6 ± 12.1/6	Head	89	Severe trauma mechanism	209	15	4.4			8
								Face	45	Motor vehicle accident	186					
								Thorax	52							
								Abdomen	28							
								Spine	29	Fall	79					
								Upper extremity	48							
								Lower extremity	10	Minor trauma mechanism	81					
								Vessels	6	Others	25					
Yamamoto, 2023 [42]	WBCT	7832	646	7186	457	68	20.79			Blunt	7738	26	23	0.0125	0.0157	8
Harntaweesup, 2022 [43]	Pan-scan	37	22	15	31	45	24	Head	1			10	15	0	0.0909	7
								Face	4							
								Pelvis	3							
Ozcete, 2022 [51]	WBCT	781	536		536	43		Head and face	157					0.015		7
								Vertebral column	127							
								Chest	202							
								Abdomen	81							
								Bony pelvis	61							
Murao, 2021 [37]	WBCT	671	463		463	51	26	Head	477	Motor vehicle accident	370			0.170		8
								Face	11	Fall	245					
								Chest	351							
								Abdomen	126							
								Extremities	232	Crushed between objects	16					
										Other	40					
Treskes, 2021 [31]	iTBCT	928	456	472	709	42	22	Head	224	Fall	283					8
								Chest	198	MVA	519					
								Abdomen	44	Other	107					
								Extremities	125							
Treskes, 2020 [28]	iTBCT	220	220		168	37	18	Head	90							8
								Chest	75							
								Abdomen	20							
								Extremities	55							
Treskes, 2020 [30]	WBCT	1083	824		824	43	20	Head	465	Fall	348					8
								Chest	435	MVC patient as occupant	391					
								Abdomen	116	Patient as cyclist	125					
								Extremities	304	Patient as pedestrian	74					
										Other	126					
Treskes, 2019 [29]	iTBCT	172	85	87	135	41	27	Head	37			23	20	0.129	0.241	8
								Chest	52							
								Abdomen	27							
								Extremities	62							
Yoong, 2019 [14]	WBCT	337			246	42		Head and neck	154	Fall	115					7
								Face	64							
								Chest	241	Car accidents	208					
								Abdomen	142	Crush	8					
										Blast	5					
Nummela, 2018 [38]	WBCT	1461				47.2		Chest fractures	574	Car accidents	39					7
								Costochondral fractures	221	Fall	32					
Palm, 2018 [39]	WBCT	16,928	11,307	5621	12,307	46.6	23.9			Blunt	16,132			0.157	0.159	7
										Other	796					
James, 2017 [35]	WBCT	426	220	206	285	48.8 ± 21.5		Brain cerebrovascular injury	32	Blunt		5.2	3.9	0.08	0.04	7
Treskes, 2017 [27]	iTBCT	1083	541	542	824	42	20	Head	247	Fall	348					8
								Chest	229	MVA	590					
								Abdomen/pelvic content	49	Other	126					
								Pelvis and extremities	150							
Kroczek, 2017 [49]	WBCT	2440	1735		1735	45.1	9.3	Head and face	1055							9
								Neck	235							
								Thorax	1118							
								Abdomen	2314							
								MSK	718							
Lang, 2017 [44]	WBCT	13564	8559	5005	9970	45.7	22	Thoracic injuries	75			23.3		0.156	0.155	8
								Pneumothoraces	14							
								Pulmonary contusions	21							
Hong, 2016 [46]	WBCT	144	55	89	102	42.08	35.54	Head	4			21.21 ± 20.6	25.64 ± 20.25	0.236	0.291	9
								C-spine	1							
								Chest	7							
								Abdomen	9							
								Pelvic bone	2							
								Spine	1							
								Major vessel	1							
								MSK	3							
Shannon, 2015 [50]	WBCT	588						Head and face	158							6
								Neck	76							
								Chest	278							
								Abdomen	168							
Fakler, 2014 [48]	WBCT	534	534		377	48 ± 19.9		Head	30							8
								Neck	16							
								Thorax	54							
								Abdomen	129							
								Pelvis	32							
Sierink, 2014 [26]	iTBCT	304	152	152	107	43.91 ± 19.67	18	Head	135			9	8.5	0.158	0.151	8
								Chest	71							
								Abdomen	23							
								Extremities	65							
Hsiao, 2013 [5]	WBCT	660	562	98	465	45.2	20.9			Fall	234	8 ± 22	2 ± 4	0.031	0.012	8
										Motor vehicle accident	242					
										Assault	61					
										Penetrating	28					
										Other	44					
Huber-Wagner, 2013 [25]	WBCT	9233	9233		6743	45.2	29.7 ± 12.2	Head	5229			26.4 ± 27.5	26 ± 28.4	0.174	0.214	7
								Thorax	5873							
								Abdomen	2034							
								Extremities	3760							
Kimura, 2013 [36]	WBCT	5208	5208		3698	48	26	Head	1431	Car accidents	2844					8
								Face	43							
								Neck	6							
								Chest	720	Fall	1995					
								Abdomen and pelvis	138							
								Spine	173							
								Upper extremities	56	Others	370					
								Lower extremities/pelvic ring	360							
Rosi Junior, 2012 [52]	WBCT	355						Head, neck, and face	83							7
								Spine	202							
Hutter, 2011 [47]	WBCT	1144	223	608	851		26.42							0.08	0.15	8
Wurmb, 2011 [41]	WBCT	318	155	163		38	27			Blunt and penetrating				0.086	0.09	9
Weninger, 2007 [45]	WBCT	370	185	185	134	43.5 ± 17.2	26.6 ± 10.3	Head/neck	116			29 ± 29.4	32.5 ± 33.3	0.17	0.16	8
								Chest	91							
								Abdomen	51							

*N* patients (entire population size within each study); *IWBCT* population is the portion of the population went through IWBCT; control population is defined as alternative/conventional modalities of diagnosis (other than IWBCT); ISS (Injury Severity Score) injury types have been broken down to more specific injuries when provided; MOI (mechanism of injury) were consolidated into fall and motor vehicle accidents when possible; LOS (length of stay (days) within the hospital) MR (risk ratio of mortality rate); NOS [34] refers to the Newcastle–Ottawa assessment scale of non-random research studies. Injuries may be more than one per patient

**Table 2:** Statistical analysis of the studied literature

	Number of studies	Prevalence	95% CI	*I*^2^ (%)
Sex				
Male	27	0.67		
Female	27	0.33		
Injuries				
Head	12	0.44	(0.28–0.60)	99.8
Neck	6	0.06	(0.02–0.09)	97.2
Face	5	0.09	(0.05–0.13)	97.1
Chest	19	0.39	(0.28–0.51)	99.8
Abdomen	16	0.23	(0.03–0.43)	99.9
Spine	6	0.19	(0.11–0.27)	99.4
Extremity	9	0.33	(0.23–0.43)	99.2
Pelvis	5	0.11	(0.04–0.18)	97.4

Number of patients are presented as prevalences by Sex

*CI* confidence interval pertaining to a 95% accuracy, *I*^*2*^ index of heterogeneity

### Length of stay in the hospital (LOS)

LOS of 33,146 patients (mean age: 44.45 ± 25.65, ISS: 21.98 ± 11.53) within the hospital was gathered from 11 studies [5, 24–26, 29, 35, 40, 42–46]. The mean LOS for patients going through iWBCT upon arrival to the hospital was 19.26 ± 24.88, 16 for immediate total body scan (iTBCT), and 6.9 for pan-scan. Other diagnostic techniques (MR, SCT, ultrasound, and radiograph) corresponded to a mean LOS of 16.01 ± 21.49.

### Mortality rate

The mortality rate within the hospital was gathered from 10 WBCT/pan-scan studies and 1 TBCT with a total of 57,680 patients (mean age, 46.99 ± 28.79; ISS, 25.0 ± 12.2) [5, 26, 29, 35, 39, 41–44, 46, 47]. The mean value for patients undergoing WBCT/pan-scan immediately after admission to the hospital was 12.37% and 14.35%, respectively. In contrast, other diagnostic techniques corresponded to a mean mortality rate of 14.10%. The sample size was more significant for the WBCT (34,427) than non-WBCT/pan-scan (23,310). A mortality odd ratio of 0.94 (95% CI, 0.83–1.06; *I*^2^ = 40.1%) was calculated to compare people who underwent WBCT/pan-scan and those not using these imaging scans (Fig. 9).

### Whole-body CT/pan-scan findings

A cumulative total of 33,790 injuries were found using the studied modalities of WBCT. After presentation to the emergency department (ED) within the studies, these injuries were broken down and reported based on differing body regions. Data from these studies were pooled into head/neck and chest, respectively. Overall, thoracic injuries were the leading immediate WBCT/pan-scan diagnosed injury, followed by abdominal, head, neck, face, and extremity injuries (thorax 11,028; head, neck, and face 10,516; abdomen 5600; extremities: 5203). The remaining were other injuries within the studies. Findings were then gathered further region-specific to provide a specific analysis of each’s prevalence in traumatic injury WBCT/pan-scan findings.

#### Head injuries

Twelve studies were filtered based on their presentation of data on head findings [25–27, 29–31, 36, 37, 43, 46, 48]. 14,168 patients with a mean age of 43.65 ± 19.67 years and an ISS of 24.2 were included. Our analysis found that 0.44 (95% CI, 0.28–0.60; *I*^2^ = 99.8%) of patients had head injuries diagnosed by WBCT/pan-scan (Fig. 2).

#### Neck injuries

Six studies were filtered based on their presentation of data on neck findings [36, 43, 46, 48–50]. 8,417 patients with a mean age of 45.05 and an ISS of 23.71 were included. Our analysis found that 0.06 (95% CI, 0.02–0.09; *I*^2^ = 97.2%) of patients had neck injuries diagnosed by WBCT/pan-scan (Fig. 3).

#### Facial injuries

Five studies were filtered based on their presentation of face findings [14, 36, 37, 40, 43]. 6543 patients with a mean age of 39.46 and an ISS of 21.65 were included. Our analysis found that 0.09 (95% CI, 0.05–0.13; *I*^2^ = 97.1%) of patients had face injuries diagnosed by WBCT/pan-scan (Fig. 4).

#### Thoracic injuries

Nineteen studies were filtered based on their presentation of data on the chest. Their inclusion accounted for a total of 39,710 patients. Mean age was 42.28 ± 18.92 [14, 25–31, 36–38, 40, 44–46, 48–51]. The mean ISS was 22.36 ± 11.53. Our analysis found a 0.39 (95% CI, 0.28–0.51; *I*^2^ = 99.8%) prevalence of chest injuries using WBCT/pan-scan (Fig. 5).

#### Abdominal injuries

Sixteen studies were filtered based on their data on abdominal injury WBCT/pan-scan findings. This totaled 20,226 patients [14, 25–31, 37, 40, 45, 46, 48–51]. The mean age was 41.74 ± 18.92. ISS was 22.25 ± 11.53. The meta-analysis found a prevalence of 0.23 (95% CI, 0.03–0.43; *I*^2^ = 99.9%) abdominal injury findings using WBCT/pan-scan (Fig. 6).

#### Spinal injuries

Data on traumatic spine injuries was pooled from six studies [36, 40, 44, 46, 51, 52], totaling to a group size of 20,267 patients with a mean age of 37.00. The mean ISS was 19.53 ± 12.1. Spinal injuries were calculated to have a prevalence of 0.19 (95% CI, 0.11–0.27; *I*^2^ = 99.4%) through our analysis (Fig. 7).

#### Extremity injury

Data on extremity injuries were pooled from nine studies [25, 26, 28–31, 37, 46, 49], totaling to a group size of 15,195 patients with a mean age of 41.6. The mean ISS was 43.37 ± 12.2. The prevalence of upper and/or lower extremity injuries diagnosed by WBCT/pan-scan was calculated to be 0.33 (95% CI, 0.23–0.43; *I*^2^ = 99.2%) through our analysis (Fig. 8).

#### Pelvis

Data pertaining to pelvic injuries were pooled from five studies [43, 46, 48, 51, 53], totaling to a group size of 2579 patients with a mean age of 44.02 ± 19.9. The mean ISS was 26.51. Pelvic injuries diagnosed by WBCT/pan-scan were 0.11 prevalent (95% CI, 0.04–0.18; *I*^2^ = 97.4%) through our analysis (Fig. 9).

####  Injuries Missed on non-WBCT

In four studies, missed injuries were studied in conventional imaging modalities that were later confirmed using WBCT/pan-scan, leading to a delayed course of treatment and increased morbidity/mortality [14, 24, 45, 46]. Weninger et al. and Yoong et al. provided the injuries as a definite area of missed injuries, while Hong et al. and James et al. found smaller studies identified each missed injury. An amalgamation of their data resulted in a dataset corresponding to a patient size of 1277 with a mean age of 44.8 ± 19.35. The mean ISS was 31.1 ± 10.3.

In addition to their primary data, two studies examined cases in which immediate WBCT/pan-scan findings were compared against selective CT (SCT) and whole-body MRI (WBMR). Kimura et al. [36] provided immediate SCT cases, while Raimann et al. [40] focused on WBMR. A total of 4456 injuries were found in selective CT, while WBCT found 2927 injuries within a controlled timeline in a Trauma Center in Japan. In contrast, WBCT found 307 injuries, while WBMR found 272 injuries within a controlled timeline in a trauma center in Germany.

Missed injuries in WBCT consisted of 3 bowel injuries, 1 internal iliac artery injury, 2 femur neck fractures, 4 rib fractures, 1 sub-capital femur fracture, 1 intertrochanteric femur fracture, 1 subtrochanteric femur fracture, 1 nasal bone fracture, 1 orbital roof fracture, 1 temporal bone fracture, 11 radial fractures, 10 carpal fractures, and 16 phalangeal fractures. This observed pattern of increased missed extremity injuries was attributed to the technique within the papers.

### Mortality rate analysis

Odds ratio analysis performed from the data presented on mortality rates in 11 studies demonstrated an odds ratio of 0.94 (95% CI, 0.83–1.06, *I*^2^ = 40.1%). This initially did not represent a statistically significant report (*P* = 0.082) (Fig. 10).

### Sensitivity analysis

The sensitivity analysis results revealed that any single study or cluster of studies with shared characteristics had minimal influence on the effect size and its corresponding 95%CI, indicating robustness in the findings. Sensitivity analysis rejected the null hypothesis of a single study or any cluster of studies with statistical outlier characteristics. All studies had minimal effect on effect size and 95% CI, confirming the robustness of the findings overall (Fig. 13).

### Publication bias

Figure 12 shows Begg’s funnel plot based on applying WBCT in trauma patients into body regions. The interpretation of our Begg’s funnel plot (*P* = 0.484) and Egger test (*P* = 0.193) shows no publication bias in the included studies. Therefore, it is understandable that reports have been published with both positive and negative outcomes (Figs. 11 and12).

## Discussion

While former studies on WBCT and pan-scan have looked at the prevalence of trauma and effectivity, our study takes a wider stance, taking a different approach from prior research like those conducted by Arruzo et al. [20], Hassankhani et al. [54], and Tsutsumi et al. [55]. To the best of our knowledge, our study is the first of its kind to evaluate data from WBCT/pan-scan in trauma. Our investigation casts a broader net, incorporating findings from 27 distinct studies and a vast pool of 64,924 patients for analysis. This scope enhances the robustness of our conclusions, presenting a more comprehensive and nuanced understanding of trauma prevalence.

This meta-analysis examines the relationship between diagnostic accuracy, radiation dose, and patient outcomes in trauma patients who undergo WBCT/pan-scan. It provides insights into the complex factors that influence decision-making in clinical practice. Our study found a significant difference in overall mortality and a significant reduction in hospital stay time with different WBCT implementation compared to non-whole-body approach, a notable implementation in changing trauma outcomes. The potential benefits of immediate whole-body scanning upon admission are further highlighted, given that they outweigh the increased financial cost and radiation risk when adjusted [31].

Shifting the focus to trauma prevalence, in the systematic review, a critical examination of CT utilization revealed similar patterns in trauma prevalence between studies. In trauma assessment, the study conducted by Hong et al. offers valuable insights into critical regions that demand focused attention [46]. This study emphasizes the importance of timely diagnosis and management in regions such as head, neck, and spine, including potential head bleeds and spinal injuries. Similarly, the frequency of occult traumatic chest injuries is confirmed by the results of our study, accentuating the necessity for dedicated focus in addressing thoracic injuries. The recognition of the delicate nature of this area reinforces the importance of precise evaluation and intervention. Moving to abdominal injuries, the acknowledgment of potential internal bleeding’s significant risk to mortality necessitates a distinct consideration to elucidate prevalence in this domain.

As such, looking into the data focusing on head, neck, and face injuries, our findings exhibited a substantial prevalence of 0.44, 0.06, and 0.09, respectively, underlining the efficacy of WBCT in detecting injuries in these crucial regions. The prevalence of chest injuries stood at 0.39, emphasizing the utility of WBCT/pan-scan for a comprehensive chest injury assessment. A similar trend emerged in the analysis of abdominal injuries, revealing a prevalence of 0.23. These insights underscore the importance of WBCT/pan-scan in diagnosing injuries within these diverse anatomical landscapes, providing a holistic perspective for trauma evaluations. Beyond these, our study extends its focus to spinal injuries 0.19, extremity injuries 0.33, and pelvic injuries 0.11. These results underscore the pivotal role of WBCT/pan-scan in minimizing the risk of missing injuries across a spectrum of anatomical domains, advocating for its judicious incorporation into trauma assessment protocols.

A separate and significant finding of our study was the decrease in mortality rate in WBCT/pan-scan patients compared to other diagnostic approaches. Interestingly, it has been shown in previous studies that the WBCT/pan-scan group had a higher ISS mean than the non-WBCT group, given that ISS is a strong predictor of mortality rate [56]. While this significant finding casts a light of assurance on the benefits of WBCT/pan-scan, other studies discuss other nuanced factors that have also been proven to reduce mortality rates and hospital stay time within emergency trauma patients [57–60]. Some of these factors including diagnostic accuracy, radiation dose, and patient outcomes highlight the placement of the CT device within the emergency department and other policies and clinical protocols that improve patient outcomes.

In Reske et al., radiation exposure, adherence to guidelines, and the influence of individual physician practice styles were common areas of investigation, underscoring the pivotal roles they each play in trauma management and improving patient outcomes [60–62]. In all mentioned studies, the underlying theme revolves around the evolving field of diagnostic imaging in trauma. The studies prompt a re-evaluation of protocols, emphasizing the need for tailored approaches that balance diagnostic accuracy with potential risks. When WBCT/pan-scan is not available, Kaya et al. discusses a decision-making process and intricate ways in which the effectiveness of SCT can be increased to ensure better outcomes; this involves a more elaborate understanding of the mechanism of trauma, an essential foundation of our study [56]. This analysis indicates that in trauma setting, when possible, WBCT should be given priority as the preferred imaging method. Nevertheless, when WBCT is not available, SCT may be a suitable substitute for a thorough diagnostic assessment.

Additionally, the financial implications of utilizing WBCT/pan-scan in trauma patients necessitate a level of exceptional consideration, as it is confounded with the potential alternative of repeated SCT or MRI. As discussed in Simma et al., the efficiency and speed of WBCT detection play a further pivotal role in reducing diagnostic time and minimizing the need for subsequent imaging studies [63]. The upfront cost of a single WBCT/pan-scan examination, although potentially higher than individual scans, is often outweighed by the cumulative expenses associated with repeated SCTs or MRIs, considering factors such as additional staff time, resources, and facility utilization. The streamlined diagnostic process facilitated by WBCT enhances patient care by expediting definitive treatment and contributes to the overall cost-effectiveness of trauma evaluations. This financial consideration aligns with the broader discourse on optimizing care without compromising the quality of patient outcomes, reinforcing the multifaceted advantages of WBCT/pan-scan in trauma management.

By encompassing a diverse array of studies and a substantial patient population, we hope our research can be a more accurate indicator for body-region trauma and a more accurate measure of the impact of WBCT in the trauma and emergency setting. The extensive dataset allows for a deeper exploration of trends and patterns, offering a broader perspective that contributes to the ongoing dialogue surrounding the role of WBCT/pan-scan in enhancing diagnostic approaches for trauma patients.

## Study limitations

While most studies had clear and concise definitions of protocols when referring to their techniques, we found inconsistent definitions for pan-scan or WBCT. This points out a lack of standardization and congruity among the researchers and physicians in their definitions of these protocols and, therefore, stands as a limitation of this study. At the same time, it highlights the need for a concrete set of definitions for these techniques and protocols. Further, we found a lack of concrete definitions for skull injuries, C-spine injuries, and facial injuries among several studies that we believe could be used to make further distinctions in the data in future studies.

There was a consistent lack of distinction between the reported injuries in the specific areas of the vertebral body and spine, leaving room for further investigation. Further, this study leaves out the implications of angiographical findings. While this is the case, the conclusion that the non-angiographical injuries are enough to persuade the utilization of WBCT is independent of vascular injuries, proving the usefulness of the utilization of WBCT regardless, it is worthy to note that the WBCT protocol could consist of CT angiogram of the chest and upper abdomen and CT of the abdomen in portal venous phase.

Further, we could not find more detailed information on the exact nature of pelvic and abdominal injuries in most of our reported studies. Splenic, hepatic, pancreatic, kidney, and mesenteric hematomas are only some of the critical injuries that deserve notation in the results of such a study. Due to the vast difference in the types of injury possible within these regions, we could not attribute the specificity and sensitivity of our survey to a particular tissue or injury type. Still, we believe it could be an exciting area for future research.

## Conclusion

To summarize, our study highlights the importance of WBCT in evaluating trauma patients rapidly. After analyzing data, we discovered that WBCT/pan-scan significantly aids in detecting injuries in the thoracic, abdominal, and head/neck areas. WBCT is associated with a lower mortality rate despite a hospital stay, highlighting its critical role in guiding timely interventions and improving patient outcomes.

Taken together with our findings, it is evident that the increased sensitivity of WBCT is crucial in minimizing morbidity, improving patient care in the emergency room and outpatient settings, and improving outcomes due to its high speed and availability. Further, the unquestionable application of WBCT and rare missed cases in diagnosis in the cases of head, neck, and face, thoracic, pelvic, and even abdominal traumatic injuries, amplified by the speed that it provides, allows for a level of care that is not possible, nor as comprehensive, with the use of selective imaging.

We believe that these detailed descriptions of prevalence and sensitivity are essential information for the ED physician to provide the best care for the patient and set them off on the best clinical course to remission.
